# A Robust Crowdsourcing-Based Indoor Localization System

**DOI:** 10.3390/s17040864

**Published:** 2017-04-14

**Authors:** Baoding Zhou, Qingquan Li, Qingzhou Mao, Wei Tu

**Affiliations:** 1 Shenzhen Key Laboratory of Spatial Smart Sensing and Services, Shenzhen University, Shenzhen 518060, China; bdzhou@szu.edu.cn; 2 Key Laboratory for Geo-Environment Monitoring of Coastal Zone of the National Administration of Surveying, Mapping and Geoinformation, Shenzhen University, Shenzhen 518060, China; 3 State Key Laboratory of Information Engineering in Surveying, Mapping and Remote Sensing, Wuhan University, Wuhan 430079, China; qzhmao@whu.edu.cn

**Keywords:** indoor localization, crowdsourcing, radio map, smartphone

## Abstract

WiFi fingerprinting-based indoor localization has been widely used due to its simplicity and can be implemented on the smartphones. The major drawback of WiFi fingerprinting is that the radio map construction is very labor-intensive and time-consuming. Another drawback of WiFi fingerprinting is the Received Signal Strength (RSS) variance problem, caused by environmental changes and device diversity. RSS variance severely degrades the localization accuracy. In this paper, we propose a robust crowdsourcing-based indoor localization system (RCILS). RCILS can automatically construct the radio map using crowdsourcing data collected by smartphones. RCILS abstracts the indoor map as the semantics graph in which the edges are the possible user paths and the vertexes are the location where users may take special activities. RCILS extracts the activity sequence contained in the trajectories by activity detection and pedestrian dead-reckoning. Based on the semantics graph and activity sequence, crowdsourcing trajectories can be located and a radio map is constructed based on the localization results. For the RSS variance problem, RCILS uses the trajectory fingerprint model for indoor localization. During online localization, RCILS obtains an RSS sequence and realizes localization by matching the RSS sequence with the radio map. To evaluate RCILS, we apply RCILS in an office building. Experiment results demonstrate the efficiency and robustness of RCILS.

## 1. Introduction

Indoor localization has attracted much interest in recent years due to the diverse location-based services (LBS) that require accurate positioning [1]. There are several technologies available to provide indoor positioning solutions such as WiFi [2], radio-frequency identification (RFID) [3], Bluetooth [4], Ultrawide Band (UWB) [5], inertial sensors-based localization [6,7], etc. In particular, WiFi fingerprinting has been widely used due to its simplicity leveraging on the pre-existing WiFi infrastructures. Moreover, this approach does not require any specialized hardware or additional infrastructure support because most smartphones are WiFi-enabled.

A WiFi fingerprinting-based positioning system consists of two phases: offline training phases and online positioning phases. In the training phase, a set of known locations are selected as the reference points (RPs) and WiFi Received Signal Strengths (RSSs) from all detected access points (APs) are collected at each RP. The RSSs collected at each RP are called fingerprints. To improve the localization performance, this collection takes a few seconds in every point to collect a sufficient number of measurements in order to overcome the RSS variance problem. In some other cases, the collection takes even more time when it is done in four different orientations to take into account the effect of antenna patterns [2]. After the collection, the radio fingerprint of each location is defined by averaging the RSS measurements or the statistics. The radio fingerprints of all the RPs constitute the radio map. In the online phase, the real-time RSS samples received from the APs are compared against the stored radio map to estimate the user’s location.

As can be easily inferred from the above description, building the radio map is a very labor-intensive and time-consuming process, which is the major bottleneck of WiFi fingerprinting in practical applications. To avoid a site survey, researchers have proposed many calibration-free indoor positioning systems [8,9,10,11,12,13,14]. Another drawback of WiFi fingerprinting-based localization is the RSS variance problem, which severely degrades the localization accuracy. An RSS variance problem means that the RSS vectors observed in the localization phase are different from the ones collected during the training phase. The RSS variance problem is caused by differences in device type and environmental changes between the two phases.

Crowdsourcing is the most promising solution for solving the site survey problem. This is due to the rise of the smartphone users and every smartphone user may become a potential contributor. By the built-in sensors of the smartphone, the inertial data and WiFi RSS data can be collected. Inertial data can be used to obtain relative trajectory of the user. Based on the relative trajectory, some methods are used to infer the location of each step and label the RSS vectors with the location information. Crowdsourcing is a low-cost and efficient way to extract useful information from data acquired by crowd participants. The crowdsourcing method has been successfully applied to different indoor maps and WiFi radio map construction systems [15,16]. For crowdsourcing-based radio map construction, the RSS variance problem is especially serious. The participant’s smartphones are usually different; additionally, the RSS vectors are collected at different times and in different environments.

In this paper, we propose the RCILS, a Robust Crowdsourcing-based Indoor Localization System. RCILS can automatically construct a WiFi radio map using the crowdsourcing data collected by the smartphones. Moreover, RCILS can reduce the influence of RSS variance problem by using a sequence-base radio map. RCILS is based on two key observations. The first observation is that people’s activities and trajectories in the indoor environment are restrained by the indoor map. By matching the activities and trajectories to the map, we can get the coordinates of the trajectories and label the RSS collected along the trajectories with location information. The second observation is that, during the localization process, the user is walking and the collected RSS vectors are continuous. From our preliminary experiments, we found that the changing trend of the same path at different times are similar. That is to say, due to the environmental changes, the RSS values may be different at different times, while the changing trend of the RSS during people walking along the same path changes little. Moreover, the changing trends of the RSSs collected by different types of smartphones are also similar, although the RSS values are different due to the device diversity.

The contributions of RCILS include: firstly, RCILS proposes a crowdsourcing-based WiFi radio map construction method; secondly, RCILS propose trajectory fingerprint model for WiFi fingerprint-based localization, which can reduce the RSS variance problem caused by environment changes and device diversity.

In order to realize RCILS, we propose a sequence-based fingerprint model for WiFi fingerprinting indoor localization. The sequence-based fingerprint model can overcome RSS variance problem caused by environment changes and device diversity. We represent indoor map as a semantics graph and model the radio map as the graph model. In the graph-based radio map, the edges represent the RSS sequence on the paths, and the vertexes represent the connection point of the paths. To construct the graph-based radio map, we use activity-based map matching approach to label the RSS collected during the crowdsourcing trajectories. For online localization, RCILS obtains an RSS sequence during the walking process and realizes localization by matching the RSS sequence with the radio map.

The remainder of this paper is organized as follows. Section 2 reviews the related work about crowdsourcing-based indoor localization. Section 3 introduces the methodology of the proposed RCILS. Result and analysis are in Section 4. Section 5 concludes the paper.

## 2. Related Work

WiFi fingerprinting-based localization is first proposed in a RADAR system [2], which requires a training phase and a localization phase. In the training phase, a radio map is constructed by collecting RSSs from existing APs at all the reference points. In the localization phase, location is determined by the *k*-nearest neighbor algorithm, which identifies the RSS vector that has the closet Euclidian distance to the currently observed RSS vector. WiFi fingerprinting-based techniques have been widely studied recently, and reviews are given in [17,18].

The major disadvantage of the RADAR system is that the radio map construction is very labor-intensive and time-consuming. Recently, numerous work has been proposed to minimize human effort in fingerprint training [8].

Radio map construction usually involves fingerprint collection and location labeling. For the point model, the fingerprint is collected by point-by-point manual calibration. In the point-by-point manual collection, the target area is partitioned into numerous grid cells, i.e., reference points, and then surveyors collect fingerprint samples at the center of each grids. The coordinate is the location labeling of the reference points. Typically, grids are sized between 2m×2m to 5m×5m, and dozens of samples are collected at each reference points [19]. The point-by-point manual calibration requires considerable time and effort. The walking survey was used instead to reduce the calibration effort of the point-by-point manual collection [20]. In the walking survey, the survey paths are planned in advance and the surveyors walk along the path to collect the fingerprints. The collection points do not have to be specified, and only the specific points, such as the start, corners, and the end point of the paths are marked by the surveyors. The location labeling is obtained by interpolation based on the specific points. Although the walking survey can reduce the collection effort to some extent, it still requires considerable time and effort [21]. Crowdsourcing approaches in which the fingerprint samples are collected from numerous users have been proposed to reduce the cost of radio map construction [8]. The crowdsourced samples can be viewed as unlabeled data since the true locations at which the samples haven been obtained are unknown.

Bolliger et al. proposed a crowdsourcing based radio map construction system named Redpin. In Redpin, the WiFi fingerprints are collected by user uploading [22]. Based on Redpin, Ref. [23] proposed an improved system to increase the number of available samples of the radio measurements by using an accelerometer to detect whether a device is moving or stationary. Similarly, Ref. [24] proposed an organic location system, which constructs radio map by user collaboration. In the system, users manually input their locations. Manual collection limits the application of the crowdsourcing based radio map construction system. Ref. [25] proposed a crowdsourcing based indoor localization system without manual training. In the system, the location of each RSS measurement by imposing constraints on the physics of wireless propagation model. However, it is different to get the accuracy parameters of the wireless propagation model in the complex indoor environment. Woodman and Harle [26] proposed a wearable inertial measurement unit-based WiFi fingerprints automatic construction system. The proposed system realizes pedestrian localization by combining a foot-mounted inertial unit, a detailed building model and a particle filter.

With the development of the smartphones, the built-in sensors can be used for indoor localization. Kim et al. [12] proposed a smartphone-based autonomous war-walking radio map construction system via crowdsourcing. The system used built-in accelerometer and digital compass of the smartphone to realize pedestrian localization. However, the system has the limitation that the initial location and direction need to be given. Zee [13] overcame this limitation by exploiting the constraint of the walls. Zee combined the information extracted from an indoor map and particle filter to realize pedestrian localization. During the pedestrian localization, the RSS samples of all the locations are collected and the radio map is constructed automatically.

These proposed crowdsourcing methods used a point model-based radio map, which easily suffers from the RSS variance problem caused by environment changes and device diversity. In this paper, RCILS utilizes a trajectory-based radio map model, which can improve the robustness of crowdsourcing-based indoor localization system. WarpMap also used a trajectory-based radio map model for indoor localization [27]. The difference between WarpMap and RCILS is that RCILS proposes a crowdsourcing-based radio map construction system, which uses a trajectory-based model for the data structure of radio map.

## 3. Methodology

### 3.1. Trajectory Fingerprint Model

**Figure 1:**
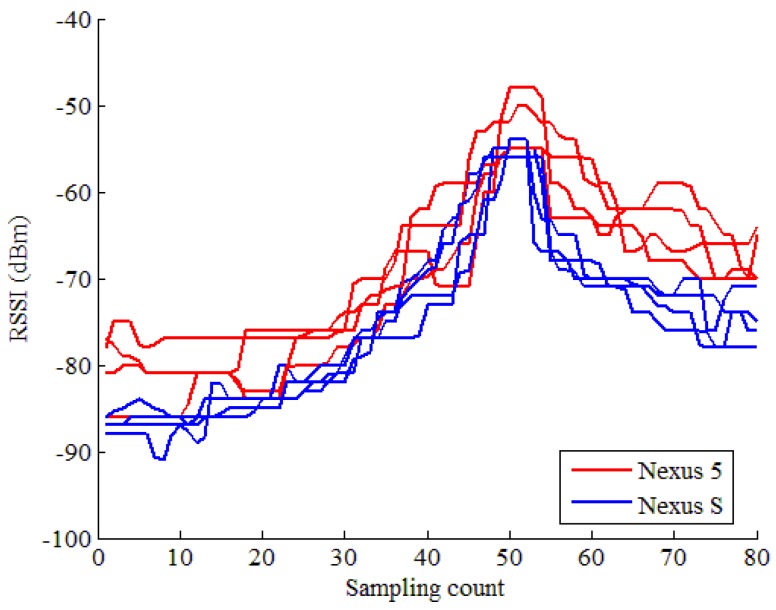
The change of the RSS (Received Signal Strength) from an AP (Access Point) during the user walking.

During experiments, we found that the change of the WiFi RSS during a trajectory is smaller than that of the fixed sampling point. We show in Figure 1 how the RSS from an Access Point (AP) changes during the user walking. The RSSs are collected by two different smartphones carried with the user. The user repeated the path four times. From Figure 1, we can see that the RSS values of different smartphones are different. For the same smartphone, the RSS values of different paths are also somewhat different. The RSS difference of two smartphones is caused by the diversity of the WiFi chipsets and antenna. The difference between different paths of the same smartphone is caused by the instability of WiFi strength. However, the changing trend of the RSSs are similar, which can be seen from Figure 1.

Based on this observation, RCILS uses a trajectory fingerprint model for indoor localization. In the trajectory fingerprint model, the radio map is stored as a graph G=(V,E). Each node v∈V is a position where a pedestrian would take special activities (special means the activities different from walking straight on level ground, including turning, taking elevator, walking stairs, etc.), and each edge $e=(v_{1},v_{2})\in E$ corresponds to a trajectory between $v_{1}$ and $v_{2}$. Besides the trajectory, the edge also includes the WiFi signatures collected when pedestrians walk along the trajectory.

RCILS includes two phases: radio map construction and trajectory fingerprint-based localization. In the first phase, the radio map is constructed automatically based on crowdsourcing data. In the second phase, RCILS realizes online localization by matching a collected RSS sequence with the fingerprints in the radio map.

### 3.2. Radio Map Construction

**Figure 2:**
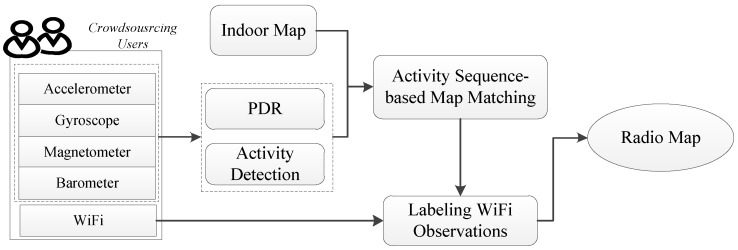
System overview of the proposed radio map construction method.

RCILS is a crowdsourcing-based indoor localization system, which utilizes built-in sensors of a smartphone to collect motion data, WiFi fingerprints and air pressure. The motion data includes acceleration, heading and angular velocity. The WiFi fingerprint includes the Medium Access Control (MAC) of the AP and the corresponding Received Signal Strength (RSS) value. The system overview of the proposed radio map construction method is shown in Figure 2.

Based on the collected data, we use an activity detection algorithm to detect the activities and use the pedestrian dead-reckoning (PDR) algorithm to estimate the distance between each two activities. The detected activities and estimated distance between each two activities constitute the activity sequence. In the proposed system, the indoor map is used as a known element. The indoor map contains useful information for indoor localization. On the one hand, it imposes hard constraints on where a pedestrian can walk. On the other hand, based on the user’s activities, the indoor map can be used to infer the user’s location. For example, if a turn activity is detected, the user may be in a corner. In this paper, the indoor map is used as a semantic graph, in which the edges are the possible user paths and the vertexes are the location where the user may take special activities. Based on the activity sequence and semantic graph of the indoor map, we use activity sequence-based matching to match the trajectory to the indoor map and get the locations of the trajectory. Then, we can label the WiFi observations based on the localization and use the labeled WiFi observations to generate the radio map.

During the online localization phase, the RSS vectors collected during the walking process constitute the RSS sequence. The length of the RSS sequence is determined by the PDR algorithm. Based on the RSS sequence, RCILS realizes pedestrian localization by matching the sequence with the sequence-based radio map.

#### 3.2.1. Semantic Graph Generation

**Figure 3:**
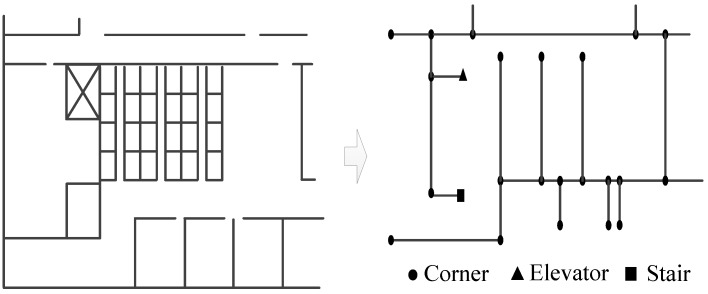
An example of semantic graph of the indoor map.

For activity sequence-based map matching, the indoor map should be converted to semantic graph, in which pathways are the edges and the intersections of the pathways are the vertexes, as shown in Figure 3. Based on the semantic graph, the location of the vertexes and displacement between each vertexes can be estimated. Moreover, the vertex also contains semantic information, which is used to match activities to the map. Figure 3 is an example of a semantic graph of the indoor map. The semantic information of the vertexes includes labelling as corner, elevator and stair.

#### 3.2.2. Trajectory Preprocessing

The trajectory of the people in the indoor map has map-related information. On the one hand, the trajectory is restrained by the topology of the map. One the other hand, based on the activity detected during the trajectory, the people’s location can be estimated. That is to say, people’s location can be estimated by matching activities to the vertexes of the graph. In order to match the trajectory to the indoor map, we should first detect the activities and estimate the displacement between each two activities.

(1) Activity detection

**Figure 4:**
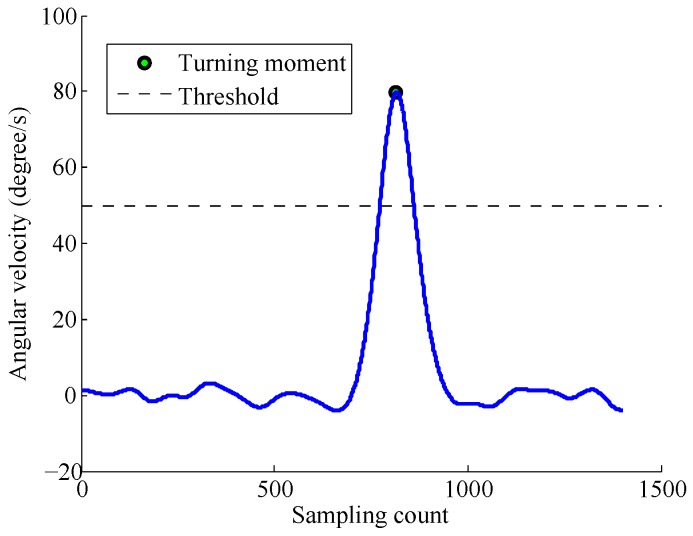
Turn detection by peak detection algorithm.

In an indoor environment, there are usually three types of activities: turning, taking the elevator, and walking stairs. Turning is the most common activity during the walking process. When a pedestrian turns, the angular velocity would generate a peak waveform, as shown in Figure 4 [16]. A turn is detected using the peak detection algorithm, which is used to find the local maximum or minimum during a period of time [28]. To eliminate the influence of the noise, a Butterworth filter of order 4 is used, with a cutoff frequency of 10 Hz.

**Figure 5:**
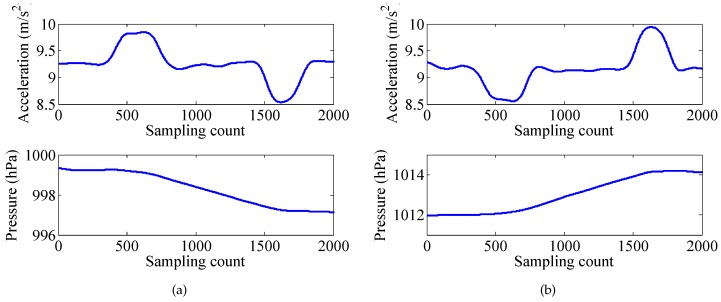
Taking the elevator. (**a**) up, (**b**) down.

**Figure 6:**
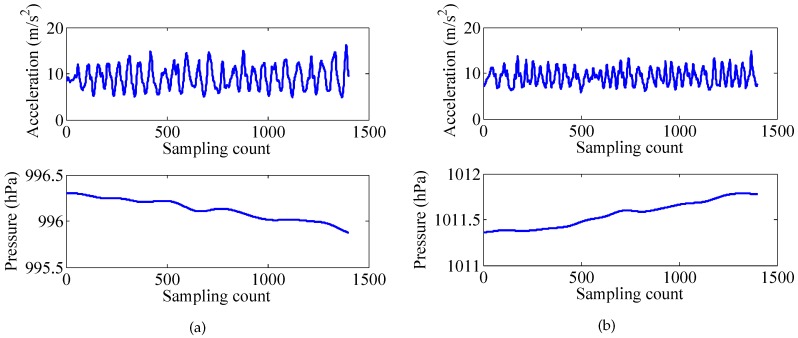
Walking up/down the stairs. (**a**) up, (**b**) down.

Generally, when the elevator rises, there will be an overweight state and a subsequent weightless state. On the contrary, when the elevator descends, there will be a weightless state and a subsequent overweight state. Moreover, the air pressure detected by the barometer can also be used for elevator detection, since the air pressure changes with the change of the altitude. The acceleration and pressure of the elevator activity are shown in Figure 5. Another activity with pressure change is walking stairs. Differently from using an elevator, during walking stairs, there is neither an overweight state nor a weightless state. The acceleration and pressure of the walking stairs are shown in Figure 6.

(2) Displacement estimation

**Figure 7:**
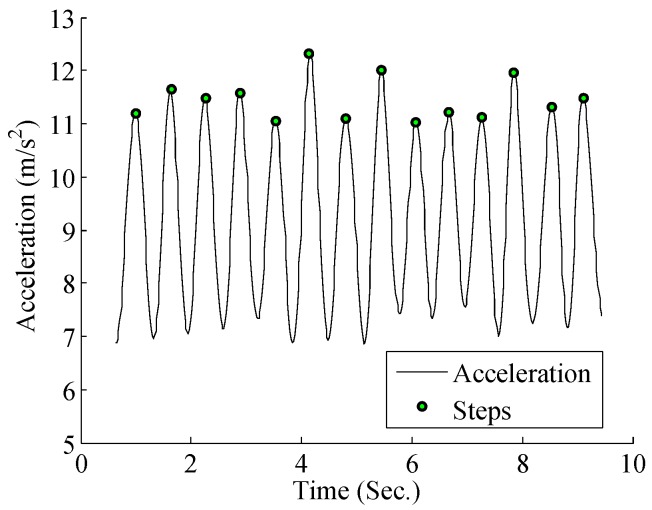
Step detection result.

The second step of trajectory pre-processing is to estimate the relative displacement between each activity. The distance estimation is implemented by PDR. PDR is a pedestrian localization scheme that estimates the relative displacement by step detection and heading estimation. Step detection is realized by the peak detection algorithm, as shown in Figure 7. When a step is detected, the location is updated by the following equation:

(1) $$\left\{\begin{array}{c} x_{t}=x_{t-1}+l\cdot \cos\left(\theta \right),\\ y_{t}=y_{t-1}+l\cdot \sin\left(\theta \right). \end{array}\right.$$

In Equation (1), $\left(x_{t},y_{t}\right)$ is the location at time *t*. *l* is the step length, calculated using the frequency-based model [29]: l=a·f+b, where *f* is the step frequency, and (a,b) are parameters that can be trained adaptively based on the matching result obtained based on activity sequence-based map matching, which is introduced in the next subsection.

**Figure 8:**
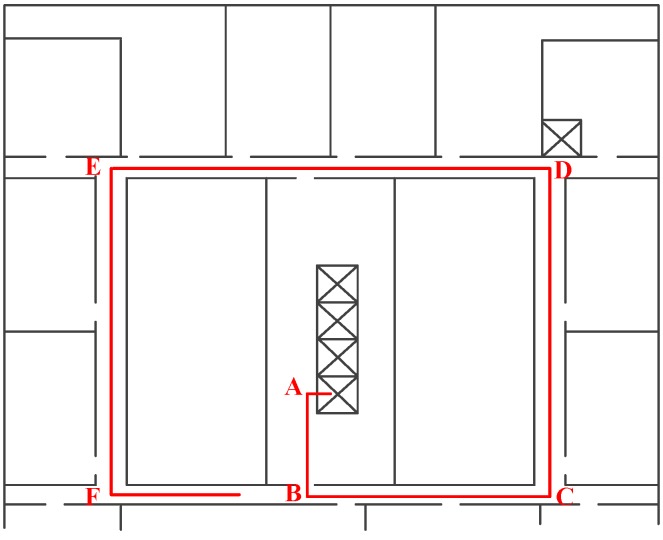
Step length parameters training.

The step length parameters is trained adaptively based on the matched trajectories. We use Figure 8 as an example to explain the parameters training algorithm. There is a trajectory which has been matched to the indoor map. Based on the known indoor map information, we can get the length of segments AB, BC, CD, DE and EF. Meanwhile, the step numbers that users walked passing these segments can be detected by the step detection algorithms. We assumed that the step length during the same segment is equal. In consequence, the step length for each segment can be calculated. The step frequency is determined based on the step detection result. The step length and step frequency for these five segments are indicated as: <L,F> = $\{\left(l_{1},f_{1}\right)$, $\left(l_{2},f_{2}\right)$, $\left(l_{3},f_{3}\right)$, $\left(l_{4},f_{4}\right)$, $\left(l_{5},f_{5})\right\}$. The parameters (a,b) are trained based on vector <L,F> using the least squares method.

#### 3.2.3. Activity Sequence-Based Map Matching

**Figure 9:**
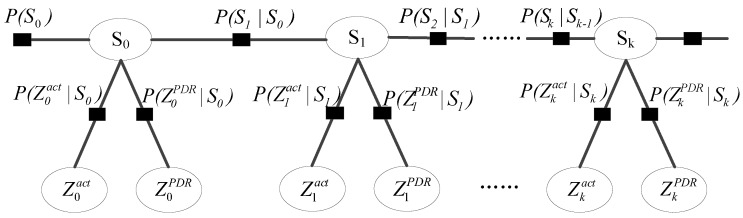
Activity sequence-based map matching method.

We use Hidden Markov Model (HMM) to match the activity sequence to the semantic graph of the indoor map. The activity sequence-based map matching method is shown in Figure 9. $S_{0},S_{1},\ldots ,S_{k}$ are the hidden state, namely the nodes of the semantic graph. $P(S_{k}|S_{k-1})$ is the transition probability from state $S_{k-1}$ to $S_{k}$. The transition is assumed to be uniform over all neighbors of a given node. The observations of the HMM are activity detection results and displacement inferred by PDR, represented by $Z_{k}^{act}$ and $Z_{k}^{PDR}$. The subscript *k* means the observations are obtained at state $S_{k}$. $P(Z_{k}^{act}|S_{k})$ and $P(Z_{k}^{PDR}|S_{k})$ are, respectively, the observation probabilities of $Z_{k}^{act}$ and $Z_{k}^{PDR}$. $P(Z_{k}^{act}|S_{k})$ describes the probability of correct activity detection for a given hidden state, namely the confusion matrix. According to the principle of PDR, $P(Z_{k}^{PDR}|S_{k})$ is made up two parts: distance observation probability distribution and heading observation probability distribution. Here, these two probability distributions are assumed to be Gaussian distributions [6,13]. Since distance and heading are independent, the observation probability distributions is defined as

(2) $$P\left(Z_{k}^{PDR}|S_{k}\right)=\frac{1}{\sqrt{2\pi }\sigma_{d}}e^{-\frac{1}{2\sigma_{d}^{2}}{\left(d_{PDR}-d_{S_{k},S_{k-1}}\right)}^{2}}\cdot \frac{1}{\sqrt{2\pi }\sigma_{\phi }}e^{-\frac{1}{2\sigma_{\phi }^{2}}{\left(\phi_{PDR}-\phi_{S_{k},S_{k-1}}\right)}^{2}}.$$

$\sigma_{d}$ and $\sigma_{\phi }$ are, respectively, the standard deviation of the distance and heading. $d_{PDR}$ is the distance calculated by PDR, and $d_{S_{k},S_{k-1}}$ is the distance between $S_{k}$ and $S_{k-1}$. $\phi_{PDR}$ is the heading estimated by PDR, and $\phi_{S_{k},S_{k-1}}$ is the angle between vector $\vec{S_{k-1}S_{k}}$ and north direction. $d_{t}$ is the distance between $z_{t}$ and the last matched state (indicated by $r_{i-1}$), $d_{i}$ is the distance between $r_{i}$ and $r_{i-1}$, $\phi_{t}$ is the angle between vector $r_{i-1}z_{t}$ and north direction, and $\phi_{i}$ is the angle between vector $r_{i-1}r_{i}$ and north direction.

Given the detected activity sequence, activity sequence-based map matching aims to find all nodes where the user completes the activities in the activity sequence. The nodes constitute the trajectory. For an activity sequence, there may be many trajectory candidates in the map. We find the best-matching one by the following equation:

(3) $$P\left(S_{k}\right)=P\left(S_{k-1}\right)\cdot P\left(S_{k}|S_{k-1}\right)\cdot P\left(Z_{k}^{PDR}|S_{k}\right)\cdot P\left(Z_{k}^{act}|S_{k}\right),\;1\leq t\leq T$$

By activity sequence-based map matching, we get the tracking results of the crowdsourcing trajectories, namely the locations where the WiFi RSS vectors are collected. Then, we can use these trajectories and RSS vectors to construct the radio map of the indoor environment.

#### 3.2.4. Radio Map Construction

The radio map is stored by the graph structure, G=(V,E,F), where *V* represents the vertexes, *E* represents the edges, and *F* represents the RSS vectors on the edges. By activity sequence-based map matching, the trajectories collected by crowdsourcing can be matched to the semantic graph. The activities contained in the trajectories are matched to the vertexes *V* of the radio map graph, and the RSS vectors collected on the edges *E* constitute the RSS vectors *F*.

### 3.3. Trajectory Fingerprint-Based Localization

In the online localization phase, the target smartphone collects RSS vectors from the surrounding APs. Moreover, by the inertial sensors of the smartphone, the moving distance can be estimated by PDR. Based on the moving distance, we generate a RSS sequence and realize localization by matching the RSS sequence with the radio map graph.

#### 3.3.1. RSS Sequence Generation

During the moving process, we get an RSS sequence with the length of the moving distance. We use $S_{t}=\left(F_{t}-w+1,F_{t}-w+2,\ldots ,F_{t}\right)$ to denote the RSS sequence collected during the moving distance, where *w* is the window size and $F_{t}$ is the latest collected RSS sample. $F_{i}=\left\{\left(mac_{1},rss\left(1,i\right)\right),\ldots ,\left(mac_{j},rss\left(j,i\right)\right),\ldots ,\left(mac_{m},rss\left(m,i\right)\right)\right\}$, $mac_{j}$ and rss(j,i) are, respectively, the MAC address and RSS value of the *j*th WiFi AP. The RSS sequence can be represented by a m×w matrix, where *m* is the number of the APs and *w* is the length of the moving window. The MAC list is $\left(mac_{1},mac_{2},\ldots ,mac_{m}\right)$:

(4) $$S_{t}=\left(\begin{array}{cccc} rss(1,1) & rss(1,2) & \ldots & rss(1,w)\\ rss(2,1) & rss(2,2) & \ldots & rss(2,w)\\ \ldots & \ldots & \ldots & \ldots \\ rss(m,1) & rss(m,2) & \ldots & rss(m,w) \end{array}\right).$$

#### 3.3.2. Graph-Based Trajectory Search

Based on the RSS sequence generation during the moving window, trajectory fingerprint-based localization is to search the best-match sequence from the radio map graph and determine the location of the best-match sequence as the target’s location.

We use Breadth-First-Search to search the best-match sequence in the graph. Searching in the whole graph needs a large computational amount. In this paper, we determine the start vertex based on the similarity between the AP list of $S_{t}$ and that of the vertex. We use the Jaccard similarity coefficient as the similarity parameter. The Jaccard coefficient is a statistic used for comparing the similarity and diversity of sample sets, which has been used for WiFi-based clustering in [16]. The Jaccard similarity coefficient is calculated using the following equation:

(5) $$J\left(MAC_{t},MAC_{i}\right)=\frac{MAC_{t}\cap MAC_{i}}{MAC_{t}\cup MAC_{i}},$$

where $MAC_{t}$ is the MAC of the AP list of $S_{t}$, and $MAC_{i}$ is the MAC of the AP list of vertex *i*. After determining the first vertex, we conduct Breadth-First-Search *c* steps to find the best-match sequence, where *c* is the constant, set to 3 herein.

#### 3.3.3. Localization

Trajectory Fingerprint-based localization is to find the best-match sequence based on the collected RSS sequence. During the graph-based trajectory searching, we calculated the similarity metric between RSS sequence and RSS in the radio map graph (called candidate RSS sequence). From Figure 1, we can see that the RSS values of different paths are different, even for the same smartphone. Therefore, using the RSS value as the similarity metric may cause localization error. In this paper, we use the correlation coefficient as the similarity metric. As before, we use $S_{t}$ to denote the RSS sequence collected during the moving distance, as shown in Equation 4. There are *m* APs in $S_{t}$, for each AP, we calculate the similarity metric, and use the sum of these metrics as the similarity between $S_{t}$ and the candidate RSS sequence:

(6) $$P=\sum_{i=1}^{m}\frac{cov(RS_{i},RC_{i})}{\sigma_{RS_{i}}\cdot \sigma_{RC_{i}}},$$

where $RS_{i}$ is the RSS set in the collected RSS sequence of the *i*th AP, and $RC_{i}$ is the RSS set in the candidate RSS sequence of the *i*th AP:

$$\begin{array}{cc} RS_{i} & =\{rss_{s}\left(i,1\right),rss_{s}\left(i,2\right),\ldots ,rss_{s}\left(i,w\right)\},\\ RC_{i} & =\{rss_{c}\left(i,1\right),rss_{c}\left(i,2\right),\ldots ,rss_{c}\left(i,w\right)\}. \end{array}$$

For the locations with null reading from the AP, −100 dB was used as the RSS value.

For each candidate RSS sequence, we get a similarity coefficient by Equation (6). We use the k-nearest neighbour (knn) algorithm to determine the best-match sequence and use the location of the terminal as the localization result. In our experiments, we set the k equal to 1 in the knn algorithm.

## 4. Evaluation

### 4.1. Experiment Setup

**Figure 10:**
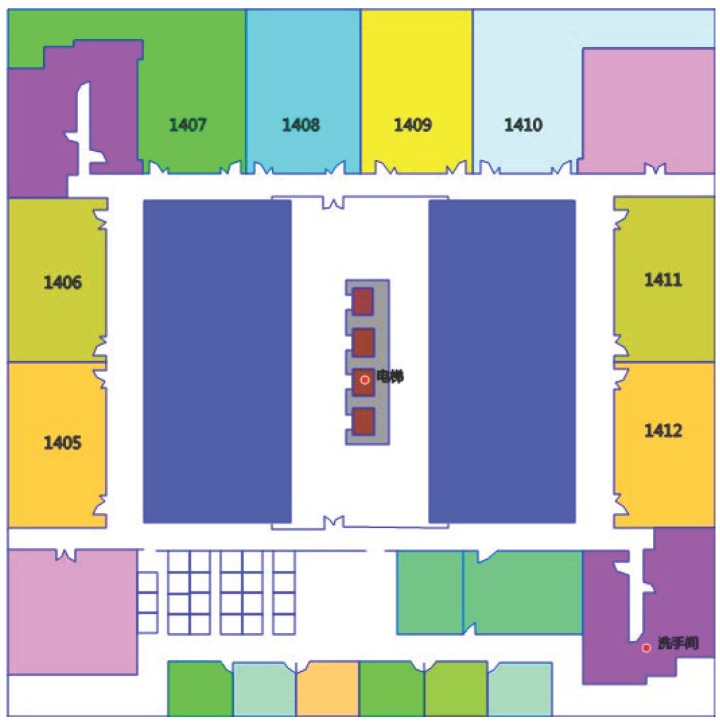
Experimental environment.

To evaluate RCILS, we performed experiments in an office building, with a 52.5m×52.5m floor plan, as shown in Figure 10. We used two different types of Android smartphones, namely Nexus S and Nexus 5, to collect the trajectory data. During the experiment, participants held two smartphones on their two hands in front of themselves and walked normally in the accessible areas of the building. Holding the two smartphones on their two hands causes the WiFi RSSs to be received at the same time. To simulate the crowdsourcing users, participants started at different positions. To evaluate the performance with incremental data, each trace is repeated ten times. In total, 200 user trajectories were collected by three participants using two types of smartphones. In terms of time, these trajectories correspond to 220 min of data collection. The collected data includes acceleration data, compass data, gyroscope data, barometer data, and WiFi.

### 4.2. Performance with Incremental Data

**Figure 11:**
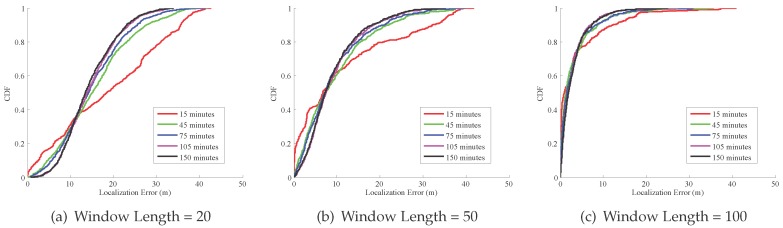

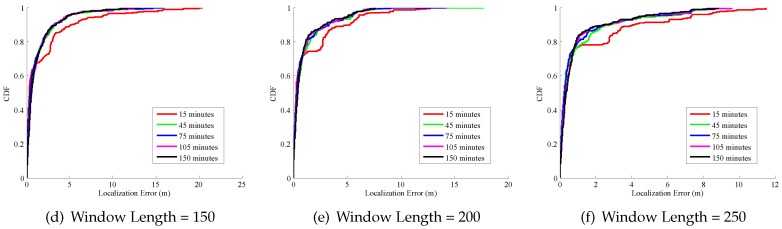
Localization error with incremental data.

The Cumulative Distribution Function (CDF) of localization error with incremental crowdsourced data is shown in Figure 11. We set different lengths for sliding windows, namely 20 samples, 50 samples, 100 samples, 150 samples, 200 samples, and 250 samples. We can see that, as the crowdsourcing data amount increases, the localization error decreases. When the length of the sliding window is 50 samples, for 15 min data, the 80 percentile of localization error is about 21 m, and when the data amount increases to 45 min, the 80 percentile of localization error decreases to about 15 m. The localization error decreases sharply when the data amount increases from 15 min to 45 min. However, when the data amount is more than 45 min, the decline of the localization error becomes smaller as the data amount increases.

### 4.3. Performance with Length of Sliding Window

**Figure 12:**
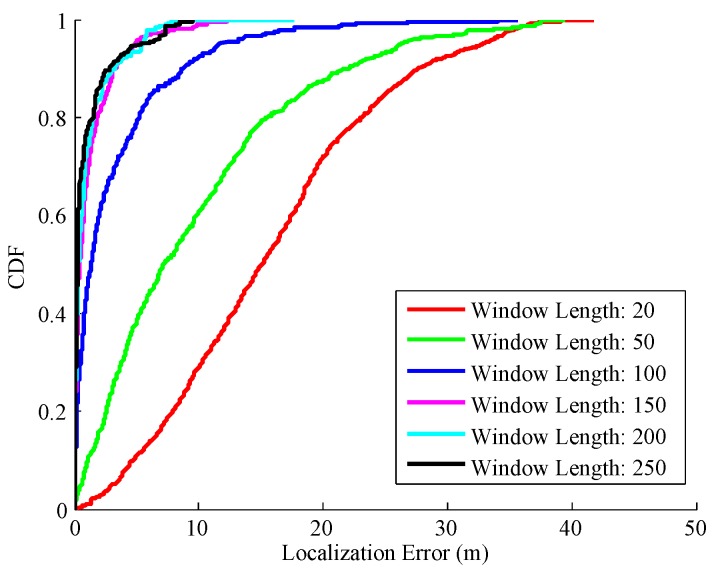
The CDF of localization error with different lengths of sliding windows (the data amount is 45 min).

Given the fixed data amount (data amount is set to 45 min), Figure 12 shows the CDF of localization error in different lengths of sliding windows. It can be seen that, with the increase of the length of the sliding window, the localization error decreases. When the length increases from 20 to 250, the 80 percentile of localization error decreases from 23 m to 1.3 m.

**Figure 13:**
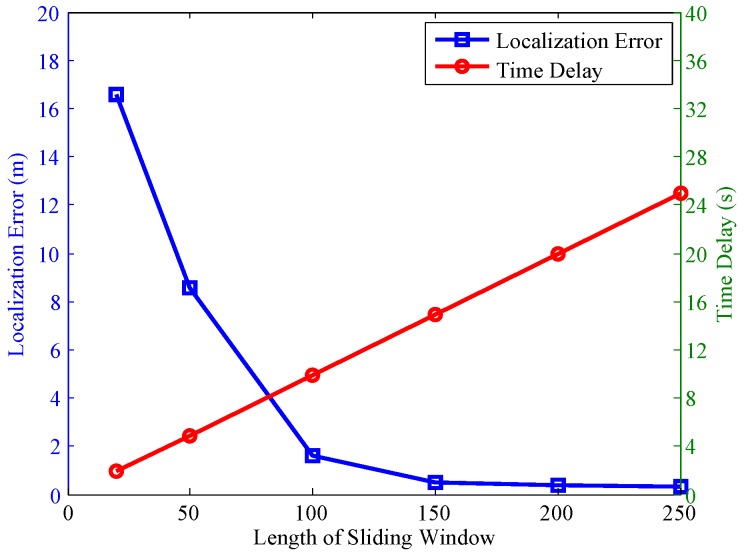
Localization error and time delay with different lengths of sliding windows (the data amount is 45 min).

The mean localization error and time delay with different lengths of sliding windows is shown in Figure 13. From Figure 13, we can see that with the increase of lengths of sliding windows, the localization error decreases. However, the long length of sliding window means a long time delay of the localization system. As it can be seen from Figure 13, when the length is 20 samples, the time delay is 1.9 s, and when the length increases to 250 samples, the time delay is 25 s.

Figure 13 shows the tradeoff between localization error and time delay with the increasing of length of sliding window. From Figure 13, we can see that the time delay increases in linear proportion to the length of sliding window. However, the downtrend of the localization error becomes smaller and smaller as the length of sliding window increases. For an online localization system, we must get a good tradeoff between localization error and time delay. For example, we can set the length to 100, and the time delay at the beginning of the system startup is 9.9 s, and the localization error is 1.6 m. Certainly, after the first localization process (i.e., the first 100 samples), the localization system does not need a startup process, and it can use the scanned samples for localization. That is to say, the proposed system just needs one time delay process at the first startup. For the offline tracking system, the time delay can be ignored, and we can use the longest length of the sliding window.

### 4.4. Performance of Different Fingerprint Models

**Figure 14:**
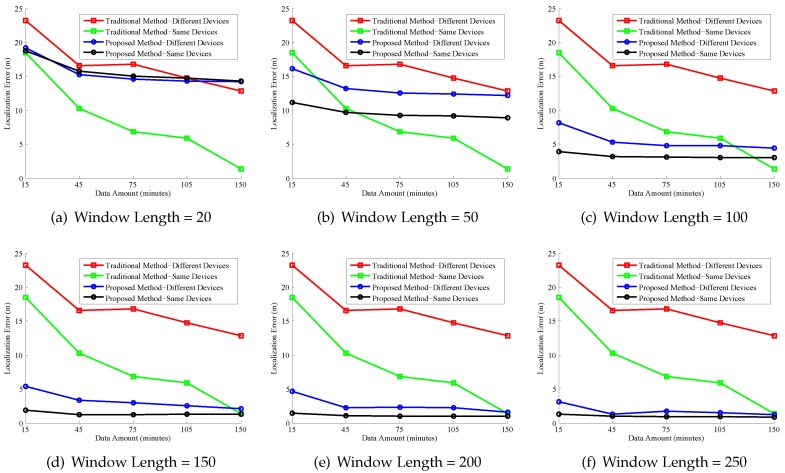
Localization error of different methods.

We compared the proposed method with the point fingerprint model. In the point model, the radio map consists of fingerprints at each reference point. The localization error of different fingerprint models is shown in Figure 14. We evaluate radio map construction method from the following two aspects: crowdsourcing data amount and device diversity.

From Figure 14, we can see that with the increasing of the data size, the localization error of the two methods decreases. However, the data amount needed for the proposed method is much more than that of the traditional method.

For the same device, if the window length is set to 150, in order to achieve 2 m localization error, the data amount needed for RCILS is 15 min, while that for the traditional method is 150 min. RCILS needs smaller amounts of data for crowdsourcing-based localization. This demonstrates that RCILS is more applicable than the traditional crowdosurcing-based system using a point-based radio map model.

Moreover, we evaluate the impact of device diversity to RCILS. In the evaluation, the data used for radio map construction and that used for online localization are different. This is common in the crowdsourcing systems since the smartphones used by the crowdsourced participants are usually different. From Figure 14, we can see that the localization error of the proposed method is much smaller than that of the traditional point-based radio map. The result demonstrates that the proposed RCILS can reduce the device diversity problem for the crowdsourcing-based indoor localization system.

### 4.5. Comparison with State-of-the-Art WiFi-Based Indoor Localization Systems

The localization performance of RCILS is compared with that of EZ [25], WiGEM [30], WILL [14], UnLoc [31], Zee [13] and LiFS [10], as shown in Table 1. We compare the localization methods in terms of accuracy, map requirements, anchor point, and device heterogeneity. We can see from the Table 1 that the median error of RCILS is 1.6 m (when the sliding window is set to 100), which is less than that of the other methods. The median error of UnLoc is 1.69 m, which is close to RCILS. However, UnLoc needs sufficient numbers of anchor points (e.g., occasional GPS location), which is not satisfied in many indoor environments. For device heterogeneity problem, EZ [25] and WiGEM [30] used a learning-based approach to train the model parameters, while RCILS used the trajectory-based fingerprint model to reduce the influence caused by the device heterogeneity. The localization error of RCILS is smaller than that of EZ and WiGEM. WILL [14] and LiFS [10] are two room-level localization systems, which is different from RCILS. Zee [13] is a map-assisted localization approach which leverages the topology of the map to restrict pedestrian’s trajectory based on a particle filter. However, particle filter is time-consuming and may be not suitable for online localization based on a smartphone.

## 5. Conclusions

In this paper, we propose a robust crowdsourcing-based indoor localization system. RCILS can automatically construct a WiFi radio map based on the crowdsourcing data. In RCILS, an indoor map is first converted to a semantic graph. The trajectory is preprocessed by activity detection and pedestrian dead-reckoning. By trajectory preprocessing, we get the activity sequence contained in the trajectory. Based on the semantic graph and activity sequence, we match the trajectory to the indoor map to get the location of the trajectory. That is to say, the location where the WiFi RSS is collected is determined by the trajectory matching. Then, the radio map is constructed based on the crowdsourcing trajectories. To overcome the RSS variance problem, we use a trajectory fingerprint model. The experiment results in an office building demonstrate that the proposed RCILS can reduce the variance problem caused by device diversity and environment changes.

In future work, we will include more activities in RCILS, such as opening the door, sitting in the office, and so on. RCILS is an offline system at the moment. We intend to develop an online RCILS system, in which the crowdsourcing data uploading and localization can be realized in real time.

## Figures and Tables

**Table 1:** Comparison with state-of-the-art WiFi-based indoor localization systems.

Method	Reported Accuracy	Map requirement	Anchor point	Device heterogeneity
EZ [25]	Median error ∼2 m inside small building (486 $m^{2}$) and 7 m inside big building (12,600 $m^{2}$)	No	Yes	Yes
WiGEM [30]	Median error ∼4 m inside small building (600 $m^{2}$) and 6 m inside bug building (3250 $m^{2}$)	Yes	No	Yes
WILL [14]	86% room level accuracy inside medium sized academic building (1600 $m^{2}$)	Yes	No	No
UnLoc [31]	Median error ∼1.69 m across three different indoor scenes (largest begin 4000 $m^{2}$)	No	Yes	No
Zee [13]	Median error ∼3 m inside medium sized building (2275 $m^{2}$)	Yes	No	No
LiFS [10]	89% room level accuracy inside medium sized academic building (1600 $m^{2}$)	Yes	No	No
RCILS	Median error ∼1.6 m inside medium sized academic building (2750 $m^{2}$)	Yes	No	Yes
